# Errors in Diagnosis

**Published:** 1884-05

**Authors:** 


### Errors in Diagnosis.

Doctors occasionally discover that they erred in diagnosis, when their case is opened at the autopsy; but this occurs with nothing like the frequency with which lower courts find their decisions "reversed" by the higher; so that, as between the two professions, the doctors have no reason to complain. The following item, from the *Ohio State Journal*, of Oct. 23d, is a trifle worse than the average—probably:

"The suit of C. Scott v. J. E. Moore et al. came up before Judge Evans yesterday, but was quickly disposed of. The jury was impaneled and sworn, when it was discovered that the case belonged to the chancery side of the court. The remarkable thing in connection with this case is, that the suit has been in court nearly four years, once in judgment, and six attorneys who were employed in it never discovered until yesterday the nature of the issues joined."—*Columbus Medical Journal.*

Solidified Creosote may be made by adding to three parts of it two parts of collodion. In this form it may be applied in toothache without the danger sometimes resulting from the liquid form.

